# CRASH-2 Study of Tranexamic Acid to Treat Bleeding in Trauma Patients: A Controversy Fueled by Science and Social Media

**DOI:** 10.1155/2015/874920

**Published:** 2015-09-07

**Authors:** Sophia Binz, Jonathon McCollester, Scott Thomas, Joseph Miller, Timothy Pohlman, Dan Waxman, Faisal Shariff, Rebecca Tracy, Mark Walsh

**Affiliations:** ^1^Department of Emergency and Internal Medicine, Henry Ford Hospital, Detroit, MI 48202, USA; ^2^College of Osteopathic Medicine, Des Moines University, Des Moines, IA 50312, USA; ^3^Trauma Center, Memorial Hospital, South Bend, IN 46601, USA; ^4^Section of Trauma and Critical Care, Department of Surgery, Indiana University School of Medicine, Indianapolis, IN 46202, USA; ^5^Indiana Blood Center, Indianapolis, IN 46227, USA; ^6^University of Notre Dame, Notre Dame, IN 46556, USA; ^7^Indiana University School of Medicine, Notre Dame Campus, South Bend, IN 46556, USA

## Abstract

This paper reviews the application of tranexamic acid, an antifibrinolytic, to trauma. CRASH-2, a large randomized controlled trial, was the first to show a reduction in mortality and recommend tranexamic acid use in bleeding trauma patients. However, this paper was not without controversy. Its patient recruitment, methodology, and conductance in moderate-to-low income countries cast doubt on its ability to be applied to trauma protocols in countries with mature trauma networks. In addition to traditional vetting in scientific, peer-reviewed journals, CRASH-2 came about at a time when advances in communication technology allowed debate and influence to be leveraged in new forms, specifically through the use of multimedia campaigns, social media, and Internet blogs. This paper presents a comprehensive view of tranexamic acid utilization in trauma from peer-reviewed evidence to novel multimedia influences.

## 1. Introduction

*(1) The Study*. The Clinical Randomization of an Antifibrinolytic in Significant Haemorrhage trial, also known as CRASH-2, has generated an unprecedented amount of controversy since its publication in 2010 [1]. This large, randomized controlled trial (RCT), which was spearheaded in England but performed in predominately low-to-moderate income countries without mature trauma systems, proposed that a bolus dose of 1 g of tranexamic acid (TXA) followed by a 1 g infusion over eight hours should be given to bleeding trauma patients. This trial's mechanistic rationale originated from TXA's use in elective orthopedic, cardiac, and liver transplant surgeries where its use led to a reduction of blood transfusion products. This effect of TXA, which resulted in its adoption in these operations, followed an extensive and thorough scientific process, which took years to establish. Based on this successful use of TXA in elective orthopedic trials, for example, it was mechanistically logical to assume that administration of TXA would decrease blood product use in trauma, obstetrics, and other situations of massive blood loss. It was with this rationale that the CRASH-2 trial was undertaken [1].

*(2) The Controversy*. The controversy regarding this revival of an older and inexpensive drug in the setting of trauma has coincided with a meteoric rise of social media sites not vetted by standard peer review that, when combined with the multimedia campaign by the National Health Services (NHS) for TXA's ubiquitous use in trauma, has led to a spirited controversy both in peer-reviewed scientific publications and throughout social media forums [2–9].

*(3) The Purpose of This Paper*. This paper seeks to analyze the scientific and historical underpinnings of the CRASH-2 trial and to contrast the peer-reviewed reaction to the reaction by social media and medical education sites driven by supporters in England and elsewhere. This controversy will be traced from the origins of the CRASH-2 trial in 2010 to current reviews, non-RCTs, and RCTs that have been proposed to address the well-described “knowledge gaps” of the CRASH-2 trial [10–24].

## 2. History of TXA Use Preceding the CRASH-2 Trial

(1) TXA, a synthetic lysine derivative that blocks the lysine site on plasminogen and inhibits fibrinolysis, was first described in 1966. Its first application demonstrated a reduction of menstrual bleeding in 1968. The use of TXA to reduce bleeding was described in many surgical and medical settings, which were expanded from the 1970s to include dysfunctional uterine bleeding, refractory thrombocytopenia, hemophilia, and von Willebrand's disease. Of importance was the adoption of TXA to treat cardiopulmonary bypass and liver transplantation-associated hyperfibrinolysis. In these settings, TXA was shown to reduce blood loss and the need for transfusion. Finally, TXA was shown to reduce bleeding and blood transfusion requirements without an increase in thrombosis in patients undergoing elective hip and knee arthroplasty [14, 25–27].

## 3. Findings of the CRASH-2 Trial

(1) CRASH-2 was a randomized, placebo-controlled trial that spanned 274 hospitals in 40 countries that sought to assess “the effects of the early administration of a short course of tranexamic acid on death, vascular occlusive events, and the receipt of blood transfusion in trauma patients” [1].

(2) Adult trauma (>16 years) patients with significant hemorrhage (systolic blood pressure <90 mm Hg or heart rate >110 beats per min, or both), or who were considered to be “at risk of significant hemorrhage” were eligible for the study.

(3) Randomization was governed by “the uncertainty principle.” The authors described this method stating, “patients for whom the responsible doctor considered that there was a clear indication for tranexamic acid were not randomly assigned. Similarly, patients for whom there was considered to be a clear contraindication to tranexamic acid treatment were not randomly assigned. However, when the responsible doctor was substantially uncertain as to whether or not to treat with this agent, these patients were eligible for randomization” [1].

(4) The trial included 20,211 patients with 10,096 in the TXA arm and 10,115 in the placebo arm. After subtracting those who withdrew consent and were lost to follow-up, the TXA arm had 10,060 and the placebo 10,067 patients.

(5) TXA was administered as 1 g bolus given over 10 minutes followed by an infusion of 1 g over 8 h.

(6) Primary outcome was death in hospital within 4 weeks of injury categorized as bleeding, vascular occlusion, multiorgan failure, head injury, or other. The primary outcome of overall mortality was reduced with tranexamic acid (T 14.5% versus P 16.0%, *p* = 0.0035, Number Needed to Treat [NNT] 67). The mortality due to bleeding was also found to be reduced (T 4.9% versus P 5.7%, *p* = 0.0077, NNT 119).

(7) Secondary outcomes were vascular occlusive events, surgical intervention, blood product transfusion, and number of transfused units. The results showed no statistical difference in the number of vascular occlusive events (T 1.7% versus P 2.0%, *p* = 0.084), the need for any surgery (T 47.9% versus P 48.0%, *p* = 0.79), transfusion of blood products (T 50.4% versus P 51.3%, *p* = 0.21).

(8) The authors also point out that “the trial inclusion criteria were clinical and did not depend on the results of laboratory tests” [1].

## 4. Peer-Reviewed Critique

The CRASH-2 trial has been scrutinized on a number of fronts starting soon after its release in June 2010 (Tables 1 [Table tab2] [Table tab3]–4).

(1) One of the first to question the CRASH-2 trial and its results was the military trauma surgeon, Dr. Schreiber, who warned against the ubiquitous and indiscriminate use of hemostatic adjuncts such as factor VIIa, prothrombin complex concentrate, and TXA for trauma resuscitation at the 2010 NIH Conference section on the Current Practice of Medicine for Severe Bleeding [28].

(2) The review by Cap et al. was one of the first to analyze the CRASH-2 trial, its findings, and the application of TXA use in trauma in July of 2011 in the Journal of Trauma, Injury, Infection and Critical Care [14]. The authors address several complaints that were circulating about the CRASH-2 trial. One of these complaints was the use of the uncertainty principle for randomization. They defend this method of patient recruitment stating, “[a]lthough this design may seem to introduce excessive physician discretion in determining patient eligibility, it should be clear that clinical equipoise could be the only ethical basis for enrolling patients in the study” [14]. They further explain that excluding patients in whom TXA was clearly indicated would actually decrease the study's ability to reveal a benefit from TXA, stating “the fact that a benefit was observed despite this exclusion suggests a strong treatment effect” [14]. A shortcoming of the study highlighted by the authors is the lack of laboratory monitoring of coagulation functions, which might have helped to elucidate the mechanism of TXA that is not obvious since its administration did not impact transfusion requirements. The authors also address the absence of Injury Severity Scores (ISS) of patients by arguing that it is unnecessary as it is retrospectively assigned, does not affect randomization, and does not reflect the amount of hemorrhage associated with the specific injury [14]. Cap et al. acknowledge the validity of the CRASH-2 findings stating, “this study was performed in a rigorous manner that reflected real-world clinical practice across a wide variety of settings, including austere environments”; however, while recommending implementation of TXA in the trauma protocol, they also urge “[f]urther research on possible alternate mechanisms of action and next-generation dosing regimens for TXA” [14].

(3) The MATTERs trial addressed some of the critiques proposed by Cap: Following the initial above noted comments about the CRASH-2 trial, the Military Application Of Tranexamic Acid for Trauma Emergency Resuscitation (MATTERs) trial was launched to address specific shortcomings of CRASH-2, including its use of civilian hospitals that lacked modern trauma systems, its lack of laboratory testing to determine coagulopathy, its inclusion of a small number of penetrating traumas, and, most importantly, its uncertainty concerning the need for an antifibrinolytic agent in patients where only half required transfusion and an equally small amount required surgery. Importantly, the MATTERs study sought to address several of those shortcomings by evaluating those patients with a clear need for an antifibrinolytic by including those who suffered combat-related injuries and required at least one unit of PRBCs [17]. Unlike the subtle CRASH-2 benefit, the MATTERs results were striking. The NNT was 1 : 7 in the MATTERs trial while it was 1 : 67 in the CRASH-2 trial [17]. The absolute reduction in mortality was a very slight 1.5% in the CRASH-2 trial compared to the 6.7% absolute reduction in the MATTERs trial [17]. In addition, the mechanism for survival remains unclear in the CRASH-2 trial as half of the patients did not receive blood. In contrast, in the MATTERs trial, all patients received blood and those who received TXA required less blood products [17]. In the CRASH-2 trial, TXA patients received the same amount of blood as those who did not receive the drug [17]. The question remains, why include patients who probably will not need blood products into a trial that tests the ability of TXA to reduce blood product use [24]?

(4) Department of Defense (DoD) and subsequent trials and reviews.

(a) Since February of 2013 there have been 2 reviews, 4 major RCTs, and several non-RCT studies launched to identify and address the concerns with TXA introduced in general terms by Dr. Schreiber. In the first of these reviews, the DoD priority trial into TXA, coauthored by Dr. Ken Mattox and others, the term “knowledge gap” was first employed to describe the multiple inconsistencies of the CRASH-2 trial [15].

(b) Soon after in June of 2013, the second review by Napolitano et al. in the Journal of Trauma and Acute Care Surgery meticulously outlined several major problems of the CRASH-2 trial [16]. The first problem addressed is the recruitment of patients according to “the uncertainty principle” where “patients were included if the responsible doctor was substantially uncertain about whether or not to treat with tranexamic acid” [1]. Patients with clear indications or contraindications to tranexamic acid were not randomized. This leads to the possibility of introduction of selection bias [16]. This method left more than 20,000 patients included in the study, who may or may not have needed an antifibrinolytic, especially given the fact that no laboratory measurements of fibrinolysis or coagulation were factored into inclusion criteria which were completely “clinical” [1]. The second major flaw is the fact that only approximately 5% of patients had bleeding as a cause of death with most of these occurring early in the first 48 hours following the injury. All other causes of death were the same in both groups with the most common cause of death being traumatic brain injury [1, 16].

There is also the inability to determine similarity between the two cohorts as neither injury severity scores nor shock status measured by lactate level or base deficit is reported. These are important measurements because previous research has shown an increasing likelihood of hyperfibrinolysis with an increasing ISS while base deficit has been shown to be an independent predictor of hyperfibrinolysis [16].

Another problem is the small sample size of hypotensive (SBP < 90 mmHg) (31.5%) and tachycardic (HR > 107) (48%) patients, who were the primary target populations [16].

In addition, TXA administration was not associated with reduced blood transfusion. Only 50% of patients received blood transfusions, which highlights the flaw of using the uncertainty principle as the fundamental entry criteria as it is uncertain whether any of the patients actually needed TXA.

Furthermore, no information is provided concerning the administration of other blood products, such as plasma or platelets, which further confounds the results and blurs the ability to claim similarity between the cohorts.

There is also a concern for inadequate reporting as “adverse events that were serious, unexpected, and suspected to be related to the study treatment were reported separately” and not on the outcome forms. Moreover, the study has a “difficult to believe” 100% follow-up as the trial spanned 274 hospitals in 40 countries. Finally, there was a small effect size. Although the results were statistically significant, they are not clinically meaningful as the study determined only a 0.8% absolute reduction in “death caused by bleeding” [1].

(c) Also in 2014, an ongoing RCT in Australia by Gruen et al. called the Pre-Hospital Antifibrinolytics for the Coagulopathy of Traumatic Hemorrhage (PATCH) reiterated the same complaints made by the MATTERs trial and Napolitano et al. [10, 16, 17, 22]. The PATCH trial should help clarify practical and mechanistic questions regarding the use of TXA in trauma resuscitation. Using a 7-point numeric system called the Coagulopathy of Severe Trauma (COAST) score criteria, the trial selects hypotensive patients who will probably require blood products [10]. Most of the patients from this trial will have SBP less than 100 mmHg in addition to well-documented and significant pelvic and/or abdominal injury [10]. One of the stated reasons to perform the PATCH trial is that the results of the CRASH-2 trial may not be applicable to economically developed countries where patients are treated more quickly [10]. Even the authors of the PATCH trial found the CRASH-2 trial problematic and as justification for their study they address the same points delineated by Gruen et al. [22]. The PATCH authors note that “Thrombotic complications were reported very rarely in the CRASH-2 study (PE, 0.7% of all patients; DVT, 0.4%) probably because they were not actively sought in many of the participating hospitals” [22]. In contrast, the MATTERs study showed that rates of PE and DVT among patients who received TXA were, respectively, 9 and 12 times the rates among those who did not [22]. Gruen et al. even hypothesize the mechanistic possibility that the 12- and 9-fold increased rate of DVT and PE in the MATTERs trial may be caused by TXA [10, 17, 22].

(d) Subsequently, in 2014, three trials were launched to investigate and elucidate the mechanism of TXA. The first, coming from the University of Pittsburgh, is the Study of Tranexamic Acid during Air Medical Prehospital Transport (STAAMP) trial [11]. The second is the Tranexamic Acid Mechanisms and Pharmacokinetics in Traumatic Injury (TAMPITI) trial from Washington University in Saint Louis, which will analyze coagulation and proinflammatory markers for those patients given TXA in trauma [12]. The last is the recently NIH-registered study of TXA in acute orthopedic fractures from the University of Tennessee [13]. Since early 2013, these RCT studies have been done to clarify the irregularities surrounding TXA use and to bridge the “knowledge gaps” that originated from the CRASH-2 trial.

(e) In addition to these RCTs, many smaller non-RCTs were performed to analyze the benefits and risks of TXA use in trauma. In September of 2013, Swendsen et al. published results from a retrospective multiple cohort study “to explore the effects of a treatment guideline for administration of TXA to patients with traumatic injury at a U.S. level 1 trauma center” [18]. They looked at mortality, transfusion requirement, thrombotic complications, and acute kidney injury [18]. Patients who were included were 18 years or older, had been injured in the past three hours, met triage criteria for serious injury, and also had one of the following: hypotension defined as SBP <90 mmHg, activation of massive transfusion protocol in ED, or direct transportation to the OR or IR suite [18]. 52 patients received TXA while the 74 historical controls did not [18]. Patients were analyzed based on TXA administration, but in order to better match controls with those receiving TXA, patients were further categorized based on the need to proceed directly to the OR/IR suites [18]. Of those patients who were transported directly to the OR, 46 received TXA and 47 did not [18]. This study showed a 24-hour mortality benefit among TXA recipients (4.3% versus 19.1%, *p* = 0.03), increased rate of DVT/PE (13% versus 0%, *p* = 0.012), and a trend towards more acute kidney injury (AKI) (28% versus 15%, *p* = 0.12), but no difference in blood product transfusion [18]. The authors conclude by emphasizing the need for more research, stating “further studies with a larger sample size in US trauma populations would be useful” because of the “unclear” reason why TXA use is associated with increased thrombotic complications and to further elucidate “the impact of TXA on cerebral edema… and acute kidney injury” [18]. Finally, the authors note that they “are still at a loss to reconcile our data with the elective surgery data that shows the use of TXA in elective surgery decreases blood transfusion” [18]. They emphasize that there is still much unknown about the risks and complications of TXA although its survival benefit is once again confirmed [18].

(f) Subsequently, Valle et al. reflected a more nuanced view of the CRASH-2 findings by publishing their experience with TXA use in trauma at Jackson Memorial Hospital in Miami, Florida, in the Journal of Trauma and Acute Care Surgery in June 2014 [19]. Their retrospective, observational, single-center study, which by its nature is subject to selection and surveillance biases, included 300 trauma patients who required emergency surgery or transfusion of blood products after being admitted to their level 1 trauma center [19]. 150 patients received TXA and were matched to 150 patients who did not receive TXA using propensity scores based on age, sex, traumatic brain injury (TBI), mechanism of injury, systolic blood pressure, transfusion requirements, and ISS [19]. Of these patients, 97% received transfusion, 78% required emergency surgery, 75% required both surgery and transfusion, 80% had a SBP <120 mmHG, and 29% had a SBP <70 mmHg [19]. These patients were unique as they were all in “severe traumatic shock” [19]. The surprising finding of this study was the increased Packed Red Blood Cell (PRBC) use, increased total fluid requirements, and, most interestingly, increased mortality among the TXA group when compared to the propensity matched group [19]. The authors propose a possible explanation for the increased fluid requirement based on a known side effect of TXA. Rapid infusion of TXA has been associated with hypotension. The authors propose that TXA infusion might be exacerbating hypotension in patients who are already hemorrhaging and hypotensive from traumatic injuries. This hypotension would require a larger dose of crystalloid [19]. Valle et al. conclude the article suggesting the necessity of further research including RCTs for TXA use in trauma in countries with modern trauma systems as their research suggests that TXA increases mortality in severely shocked trauma patients in settings where there is immediate access to blood products as in countries with modern trauma systems [19].

(g) Moore et al. and Harvin et al. have studied the impact of fibrinolysis on mortality in trauma patients with hyperfibrinolysis as measured by a rapid thromboelastography (rapidTEG) and defined by the percent lysis measured 30 minutes after the maximum amplitude (LY 30%) >3% [29, 30]. Harvin et al. found that TXA utilization was not associated with reduced mortality [30]. They noted that the rapidTEG is less accurate at identifying functional hyperfibrinolysis when compared to the kaolin TEG [30]. These results further support the findings of Valle et al. questioning the obligatory adoption of TXA, as advocated by the CRASH-2 investigators, in advanced level 1 trauma centers with immediate access to blood products for resuscitation and emergent operative intervention [30]. Furthermore, Moore et al. have described phenotypic patterns of fibrinolysis using the rapidTEG and noted that “exogenous inhibition of the fibrinolysis system in severely injured patients requires careful selection, as it may have an adverse effect on survival” [29].

(h) Finally, Cole et al., with CRASH-2 authors Davenport and Brohi, have recently acknowledged the “evidence gap” concerning the use of TXA in trauma in a study published in February 2015 in Annals of Surgery [20]. They recognize the limitation of the CRASH-2 trial stating that “the uptake of TXA use in civilian trauma has been variable, in part due to the difficulty in translating the results of these studies to mature trauma systems, with differences in study populations, logistics, and resource availability” [20]. This criticism has been raised many times prior to this acknowledgement [16, 17, 22]. Similar to Valle et al.'s retrospective, observational, single-center study, which may be influenced by selection and surveillance bias, Cole et al.'s prospective cohort study is subject to a similar bias of small numbers and some insignificant outcomes, which may be different with a larger number of participants [19, 20]. Cole et al. analyzed 385 trauma patients admitted with an ISS >15, and patients were separated into shock and nonshock groups based on a base deficit ≥6 mEq/L [20]. They were further divided based on TXA administration [20]. Among nonshocked patients, there was no difference in mortality among the TXA and no TXA groups [20]. Among shocked patients, the authors present different mortality rates based on the unadjusted data versus that arrived at by univariate analysis [20]. Based on the data without statistical manipulation, the authors conclude, “Unadjusted mortality rates between the 2 groups (shocked patients who received TXA and those who did not) were the same.” Concerning the shocked cohort, they add that “[e]arly mortality rates for those who had TXA were lower.” However, it should be emphasized that the unadjusted data does not show a statistically significant difference in either early or late mortality in the severely shocked patients even though the absolute number of deaths was less in the shocked patients who received TXA [20]. A result from the unadjusted data that was statistically significant was “a fourfold increase in thromboembolic events in the TXA group” among the shocked patients (TXA 8% versus no TXA 2%, *p* < 0.01), confirming the concern for increased VTE with TXA use. The authors then performed a univariate analysis of the data, concluding that “TXA was independently associated with a reduction in MOF (multiple organ failure) and mortality in shocked patients and greater numbers of VFD (ventilator free days)” [20]. These statistical findings are presented because the subtle benefits require attention to the details of the analysis. What remains important, however, is that even with the use of subtle statistical justification of their conclusions, Davenport and Brohi, in the Cole et al. study, as CRASH-2 authors, have shifted from their previous position of nearly universal use of TXA in trauma. They conclude the article by stating, “on the basis of the findings from the severely injured cohort in this study, it is difficult to recommend its use in nonshocked patients within mature civilian trauma systems” [20]. They ultimately recommend conditional TXA use, stating that “the findings give a clear signal for using TXA in severely injured, shocked civilian patients.” However, careful analysis of their data suggests that TXA use in mature trauma systems does not reduce unadjusted mortality rates, further supporting the calls for more evidence on TXA use in trauma prior to its universal adoption in protocols.

The percentage of VTE increased fourfold when comparing shocked patients who received TXA and those that did not and reflects the similar increase found in the MATTERs trial, which was absent in the CRASH-2 trial [1, 17, 20]. In this trial, VTE was looked for and “confirmed by either ultrasound scan (deep vein thrombosis) and/or computed tomographic pulmonary angiography (pulmonary embolism)” [20]. In contrast, CRASH-2 was criticized for possible “inadequate reporting” as many speculated that there would have been increased thromboembolic phenomenon had these complications been actively sought [16, 22]. This is even more likely in light of the results of a small, non-RCT study, including CRASH-2 authors, which confirmed an association between TXA and VTE that was previously noted in an orthopedic RCT using TXA in hip fracture repair [20, 31]. It should be acknowledged, however, that the increased rate of VTE in the Valle et al. and Davenport and Brohi el al. studies in the TXA treated group may reflect the increased morbidity of severely bleeding patients given TXA, which itself might predispose to higher rates of VTE development as noted in these smaller trials and the MATTERs trial [17, 19, 20].

## 5. From Science to Social Media

This controversy has been carried out not only in peer-reviewed journals as outlined above, but also in highly influential social media forums and medical education websites, beginning with the promulgation of the CRASH-2 data in multimedia outlets.

(1) The CRASH-2 trial was published in Lancet in July 2010 [1]. Dr. Roberts, one of the authors of the trial, noted in an interview that “[s]cience is good at getting at the truth, but once you've found the truth, the methods that you use with science aren't very good at getting the truth remembered. What we're trying to do is get science to find the truth and use art to remember the truth” [32]. With this rationale, he began a campaign to increase the use of TXA in trauma by turning to “more innovative than traditional methods” [32]. These efforts spanned many multimedia milieus. One use of art by Dr. Roberts came with the commissioning of his nephew to make a Claymation Youtube video depicting “TranMan,” a motorist bleeding to death after trauma, who is saved by the administration of TXA [4, 33]. On his blog, Dr. Robert's nephew claims “that a prompt injection of a low cost drug called Tranexamic Acid (TXA) could reduce death by hemorrhage by 30%. Low profit drugs, such as TXA, do not receive big advertising budgets; Dr. Roberts therefore hoped a web video would help spread the word amongst Doctors” [4]. Roberts also promoted TXA by writing lyrics for a song performed by the Barking and Dagenham Community Choir [5]. He then commissioned artist Emma Vieceli to create a comic book promoting TXA use in a “mass-casualty” event [3, 34]. The comic book plot, mirroring a soap opera, includes TXA use in victims of a bombing, a stabbing of an ED doctor, and a romance between two doctors [34]. Concerning the comic book, Roberts states, “We also tried to make doctors giving tranexamic acid look sexier than doctors not” [3]. The comic book was printed and disseminated “to all emergency departments in the UK and many overseas” [32]. Finally, because “reduction of the global burden of disease and injury is an urgent moral obligation,” the authors of CRASH-2 launched a “Trauma Promise” campaign online, inviting healthcare workers and institutions “to make a promise to their communities that they will review the new evidence on tranexamic acid and apply it to improve the care of trauma patients” [2].

(2) In complement to Roberts' efforts, many other media sources also promoted or referenced TXA. The British Broadcasting Corporation (BBC) featured TXA in its premiere of the show* An Hour to Save Your Life* while the American Broadcasting Corporation (ABC) had an episode of the* Catalyst* called “Thin Blood” focusing on TXA [6, 9]. TXA was even bolstered in the popular British medical drama,* Holby City* in Series 16, episode 24 [7].

(3) CRASH-2 was debated in not only peer-reviewed journals, but also online among medical education websites and throughout social media forums like Twitter. Advances in technology have given rise to online medical education movements such as Free Open Access Meducation (FOAM) [35, 36]. This important and particular movement started around 2011 with the appearance of blogs focusing on medical education, and the term “FOAM” was coined in 2012 by Dr. Cadogan, coauthor of the blog, Life in the FastLane [35]. FOAM consists of “a personalized, continually expanding database of resources for medical education: podcasts, blogs, videos, modules, Facebook groups and Twitter feeds” [36]. FOAM has exploded in the area of emergency medicine with a proliferation of blogs, podcasts, and education sites [37–48]. These sites have served as a medium through which CRASH-2 was also analyzed and debated. The discussion began with literature analyses on several sites such as the EMCRIT podcast with CRASH-2 author, Tim Coats, and NNT's look at TXA where they note that the CRASH-2 trial was performed in countries that “lacked [a] sophisticated trauma system,” making it “[p]ossible that the benefits of this drug would be lost in a more modern context” [49, 50]. However, NNT still recommended TXA use as the “drug is inexpensive and, based on this data, demonstrated no associated adverse effects” [50]. A piece published in October of 2013 on the Maryland Critical Care Project site entitled “How CRASH-2 got it wrong” fueled a “FOAM firestorm” as many bloggers weighed in on TXA and debated following this podcast [51, 52]. This eventually led to a rebuttal three days later entitled, “How CRASH-2 got it right” by Dr. Weingart, also posted on the Maryland Critical Care Project site [53]. Many EM and trauma physicians also weighed in on the debate through Meducation sites. For example, in 2013, Dr. Westafer, author of “the short coat” blog, reviewed and supported TXA use in trauma and noted, “many physicians do not know about the drug as the inexpensive drug lacks a marketing campaign from the pharmaceutical industry. This is where free, open access, medical education (FOAM) may have a role in the knowledge translation gap regarding TXA” [54]. In May of 2014, Dr. Carley, of the virtual “St.Emlyn's” blog, wrote, “Here at St.Emlyn's we are big fans of tranexamic acid in trauma…We honestly believe that as the evidence stands, at this moment in time, you should give it. CRASH-2 was a great trial and the evidence is there. Seriously. Use it, it's great” [55]. After stating his support of TXA, he proceeded to analyze the study by Valle et al., noting, “[n]ot everyone is convinced though. There is an RCT ongoing in Australia (PATCH trial) and a number of other groups have raised questions too about whether we should be giving it to everyone and when” [55]. In the same blog entry, Carley criticizes Valle et al. and rejects the results of that study stating that “it's not a great design, the patient selection is unrealistic of clinical practice decision making and the results are probably a function of these inherent biases,” specifically, “methodological bias” [55]. He urges the continued use of TXA in trauma stating, “I implore you please to not stop using a cheap and easy to administer drug on the findings of this study” [55]. A summary of the CRASH-2 trial with a description of the controversies that have ensued has been published on WikEM under the title “EBQ:CRASH-2 Trial” [48].

(4) Even expert researchers in trauma departed from the pages of peer-reviewed articles and discussed the CRASH-2 trial on Twitter. Participating in the lively debate was one of CRASH-2's most influential authors, Dr. Karim Brohi, whose groundbreaking research established the etiology of trauma-induced coagulopathy (TIC) as a function of activated protein C (aPC) mediated fibrinolysis. This aPC hypothesis of trauma forms the foundation of the utilization of an antifibrinolytic to treat TIC. Dr. Brohi and the Australian traumatologist, Dr. Russell Gruen, debated the necessity for further RCTs on Twitter outside the conventional peer-reviewed confines. Dr. Gruen, author of the PATCH trial, tweeted on September 13, 2013 “@karimbrohi @rfdsdoc in my view NHS jumped the gun fast tracking TXA. I'd love it 2 b a great drug for us, just do not have evidence of it yet.” Dr. Brohi replied back “@rustygroin @rfdsdoc I'm all for PATCH. More than one RCT is important. But disagree with the excuses against translation of CRASH-2 results” (available from https://mobile.twitter.com/karimbrohi/status/374505742176632832). These important discussions occurring casually and unchecked by peers via social media may have unforeseen repercussions on professionalism and productive scientific debate.

## 6. The Environment and Economics of CRASH-2

(1) The final method utilized to promote CRASH-2 was an appeal to the current topic of global warming. Subaiya and CRASH-2 author and marketing leader Roberts in their paper “Reducing the Environmental Impact of Trials: A Comparison of the Carbon Footprint of the CRASH-1 and CRASH-2 Clinical Trials” sought to audit and compare the environmental impact of the Corticosteroid Randomisation after Significant Head Injury (CRASH-1) and CRASH-2 as measured by greenhouse gas emissions and overall carbon footprint [1, 56, 57]. The authors, in compliance with England's National Institute for Health Research carbon efficiency guidelines and in collaboration with Edinburgh Centre for Carbon Management, hired an independent agency to complete an audit of the carbon use (electricity, natural gas, heating oil, flights, and air freight) in several trial activities associated with the study which included coordination center, trial-related travel, trial team commuting, and freight delivery. The authors concluded that the carbon efficiency associated with CRASH-2 was due primarily to the decreased time frame of patient recruitment, which was enhanced in CRASH-2 by the fact that recruitment was done in Asia, South America, and Africa, where the regulatory environment is less restrictive. Because CRASH-1 trial data was entered by hand by two researchers, while the CRASH-2 trial data was entered directly by computer onto spreadsheets, they calculated a net deficit carbon footprint of CRASH-2. However, perusal of CRASH-2 trial methods section reveals that many of the centers did not have phones for randomization, and therefore, the assumption is that the data had to be either sent later by regular postal mail or by fax from another phone. An important, unanswered question is whether or not the energy consumption associated with transmission of data to the coordination site in London was included in the energy audit. The inexact estimation and auditing of carbon emissions was another limitation of Roberts' energy study, which mirrors the problems with CRASH-2 methodology, especially when CRASH-2 authors express concerns for the low rate of “serious and unexpected” thromboembolic complications associated with TXA. This is further reflected in the inexplicable 100% patient follow-up. The parallel of the two studies, CRASH-2 and the “Reducing the Environmental Impact of Trials” paper, lies in the authors' self-admitted underestimation of thromboembolic complications in the CRASH-2 trial and the unintentional underestimation of carbon emissions of the CRASH-2 trial as well [1, 56, 57].

(2) The politics of the administration of TXA in trauma, both in the UK and abroad, are not limited to the concern for global warming but also extend to the realm of economics, “urgent moral obligation” as mentioned above by Roberts, and even a matter of “Social Justice” [2, 58]. The paramount study concerning “Social Justice” in medicine is the 2010 Global Burden of Disease Study which prompted Roberts et al. to use the proposed cost effectiveness of TXA to spur interest in the CRASH-2 findings and seek acceptance into transfusion protocols in trauma by making TXA more politically and socially palatable [2, 59, 60]. Roberts states, “[e]stimation of the global burden of disease and injury is a challenging scientific endeavor. Reduction of the global burden of disease and injury is an ‘urgent moral obligation.' To reduce the human and economic effect of injury, we need better prevention, effective and affordable treatments, and the tenacity to ensure their universal access. For bleeding trauma patients, we now have an effective treatment that is affordable and widely practicable…. We have the evidence- we must use it in the service of humanity” [2, 61]. Note Roberts' use of the term “urgent moral obligation” [2]. Physicians can sign up on his “Trauma Promise” website where hospitals that have allied themselves with CRASH-2 are listed, but interestingly not a single hospital listed is from the United States [2]. Thanks in large part to the multifaceted media blitz, CRASH-2 has had an influence on trauma care guidelines in numerous organizations such as the UK NHS ambulance service, the British Army, and the US Army and has even been included on the WHO Essential Medicines List [2, 60, 62]. Despite CRASH-2's lack of methodological rigor perceived by many traumatologists and the “knowledge gaps” and “evidence gaps” as noted even by the authors themselves, the political landscape that attributes immense importance to environmental causes (e.g., global warming), “urgent moral obligation(s),” and “Social Justice” has enabled the CRASH-2 findings to make their way into trauma bays [2, 15, 16, 22, 58, 61].

## 7. Mechanistic Rationale of TXA and Previous Precedents

### 7.1. Lacking Mechanistic Rationale

Lack of mechanistic rationale has been noted for TXA use in trauma resuscitation by trauma researchers in England, Australia, and the United States. Previous use of TXA in orthopedic surgery was associated with reduced blood product use suggesting a possible antifibrinolytic mechanism for TXA [20, 27]. In contrast, in the CRASH-2 trial, patients who received TXA required the same amount of blood products as those who did not receive blood, although the TXA group did have a slight mortality benefit [1]. It has been proposed that the anti-inflammatory effects of TXA may mediate this small survivor benefit [20]. However, as CRASH-2 did not include laboratory coagulation studies and included patients who did not receive transfusion, there is no simple mechanistic rationale that explains mortality reduction confined to a three-hour window after which the drug has harmful effects [16, 63]. One problem with giving an antifibrinolytic without any markers for fibrinolysis is that there is no clear clinical definition of significant fibrinolysis [64].

The clinical definition of significant fibrinolysis is not standardized [14, 16, 65]. Currently there is experimental work to predict clinically significant fibrinolysis by plasmin antiplasmin (PAP) levels [66]. However, PAP levels, as a manifestation of clinically significant fibrinolysis, may be excessively sensitive in determination of fibrinolysis much as the D-Dimer or Fibrin Split Product, rendering these sensitive tests of little use in acute trauma. Furthermore, the range of published clinically significant fibrinolysis varies widely from 3% to 34% lysis at 30 minutes using TEG/ROTEM [67–69].

Of final interest regarding the pathophysiology of TIC there has been recent evidence by Campbell and Cap, and Cap and Hunt, concerning the effect of the platelet within the context of TIC [70, 71]. They have demonstrated that activated protein C (aPC) at low concentrations associated with TIC is inactive due to preserved activity of platelet factor 5 [70, 71]. They similarly demonstrate that platelets can prevent anticoagulant and fibrinolytic effects of aPC at concentrations that have been described in patients with TIC [70, 71]. They suggest that the cause of the elevation of aPC seen in TIC may be a “downstream” marker for “robust” or “exuberant” activation of protein C [70, 71]. They suggest that aPC is not an anticoagulant in trauma since it does not prevent or weaken the clot except at extremely high concentrations not seen in trauma [70, 71].

It has been proposed that aPC is a major effector of TIC through cleavage of factors Va and VIIIa. In addition, by binding plasminogen activator inhibitor-1 (PAI-1) and derepressing tissue plasminogen activator (t-PA), it may activate fibrinolysis [72]. This mechanism is plausible but problematic due to the kinetics of the reactions. Platelets and plasma factor Va are resistant to aPC cleavage at concentrations of aPC seen in TIC or even therapeutic use of recombinant human aPC in sepsis [70, 71].

However, central volume hypovolemia induced by orthostatic hypotension and experimental syncope in normal subjects has been shown to result in increased levels of aPC, t-PA, and increased lysis as determined by thromboelastography. These findings suggest that fibrinolysis is part of the hypovolemic pathophysiologic response to shock, but these findings do not justify the use of TXA for the scientific equivalent of the common faint, for example, [73].

### 7.2. Consequences of an Unknown Mechanistic Rationale and Imposition of Large, Government Funded RCTs on Medical Practice with Subsequent Retraction

Previously, the National Institutes of Health (NIH) funded National Acute Spinal Cord Injury Study (NASCIS) promoted the indiscriminate use of methylprednisolone to patients with cervical spine injury with a prepublication printed mailing and facsimiles to most emergency physicians' homes and departments in the United States in the early nineties [74]. Subsequently, this arbitrary administration of a “harmless” dose of methylprednisolone to patients with cervical spine injury based on this NASCIS study resulted in the inclusions of methylprednisolone into countless algorithms and guidelines [75]. Similar to CRASH-2, this study was a government sponsored and financed enterprise with little mechanistic rationale that described a subtle and equivocal benefit in spinal-cord injured patients. It took more than a quarter century for this study to be refuted with the description of increased complications of severe sepsis and pneumonia in patients who received the methylprednisolone [75]. As a result, it is no longer “endorsed by major society guidelines” [76]. For fear of repeating the past mistake of the NASCIS trial by overlooking potential complications such as the thrombotic complications noted in subsequent non-RCTs by many traumatologists, including the CRASH-2 authors, TXA's implementation into trauma protocols may have been delayed [19, 20, 76]. Ironically, the CRASH-1 trial revealed the complications of steroid use in TBI, which was legitimized by previous NASCIS recommendation of steroid use for trauma-related neurologic injuries [57, 74, 77].

## 8. Summary

(1) In summary, TXA has been described in the CRASH-2 trial as a successful treatment for the reduction of mortality in hemorrhaging trauma patients. The reduction in mortality was subtle, and the mechanistic rationale was elusive because of the absence of reduced blood transfusion products in patients who benefited from the administration of the drug. This large RCT, which was based on a reasonable foundation of previously published non-RCTs and RCTs concerning the use of TXA for bleeding patients, has not been widely accepted in the trauma community due to perceived problems regarding data collection and methodology. These concerns by many respected trauma specialists have been amplified by the unprecedented multimedia, social media, and non-peer-reviewed medical and nonmedical publications by some of the CRASH-2 authors, which have advocated for the near ubiquitous use of TXA in trauma. In addition, the authors of the CRASH-2 trial have surreptitiously used the CRASH-2 data to extoll the virtues of a reduced carbon footprint in the CRASH-2 trial compared to the CRASH-1 trial by publishing these findings in a peer-reviewed medical scientific publication. They describe the “environmental wholesomeness” of CRASH-2 in a valid scientific journal that meets the standards of peer review. Such fringe concepts undermine the true utility of the CRASH-2 trial. The momentum to use steroids in neurologic injury following the NIH funded NASCIS trial is a part of history that trauma surgeons are reluctant to repeat. This history may contribute to the previously noted delay of widespread implementation of TXA in trauma [19, 20, 76].

(2) There is excellent mechanistic rationale based on previous studies concerning the use of TXA in elective orthopedic surgery, cardiopulmonary bypass, and liver transplantation that theoretically supports the use of TXA for bleeding trauma patients as proposed in the CRASH-2 trial. However, the previously mentioned multimedia campaign, social media debates, Meducation blog entries, and extra medical publications by some of the CRASH-2 authors have clouded scientific debate and created this interesting controversy that is reminiscent of a previous debate concerning the use of corticosteroids for neurologic injury that was ironically ended by the CRASH-1 trial. This current review is the first to describe for the CRASH-2 trial the history of the phenomenon of multimedia, social media, and non-peer-reviewed publications' influence on scientific discovery. This problem will become much more common with the advancement of communication technology and reliance upon non-peer-reviewed social media for medical information.

(3) A prescient warning regarding the search for a simple pharmacologic treatment for the hemorrhaging patient was delivered by the military trauma surgeon, Martin Schreiber, at the 2010 NIH Conference section on the Current Practice of Medicine for Severe Bleeding, where he stated the following: “Interestingly, and I think we learn this very, very well from the Factor VII process, the use of these drugs may not be justified based on the data that we have. And I am truly hoping that we do not go through the same process with prothrombin complex concentrate and tranexamic acid that we did with Factor VII and then we get good prospective randomized trials we find out that these drugs are not—really are not indicated. And I think the bottom line here is that that we really haven't found a way to beat that qualified trauma surgeon with the $0.50 silk suture. There are no magic bullets when it comes to drugs for stopping bleeding” [28].

## Figures and Tables

**Table 1:** Randomized controlled trials involving TXA use.

Study	Author(s)	Description	Year	Outcomes	Recommendations
CRASH-2 [1]	Shakur et al.	20,211 pts with recruitment by “uncertainty principle”	2010	All-cause mortality 14.5% tranexamic acid (TXA) group versus 16.0% placebo group, *p* = 0.0035; vascular occlusion 0.3% TXA group versus 0.5% placebo group, *p* = 0.096	Tranexamic acid should be used in bleeding trauma patients

PATCH [10]	Gruen et al.	1184 pts in shock determined by COAST criteria	July 2014 start date	Ongoing study	

STAAMP [11]	Universities of Pittsburgh, Rochester, Texas at San Antonio, and Utah	1000 pts with prehospital shock and nonshock	July 2015 start date	Ongoing study	

TAMPITI [12]	Spinella and Bochicchio	150 pts who received one blood product and/or immediate transfer to OR	Fall 2015–Spring 2017	Ongoing study	

Tranexamic Acid in Orthopaedic Trauma Surgery [13]	Kiner	100 pts with orthopaedic trauma (hip or knee)	May 2012–Dec. 2015	Ongoing study	

**Table 2 tab2:** Review articles on TXA use in trauma.

Title	Author(s)	Description	Year	Critique
Tranexamic Acid for Trauma Patients: A Critical Review of the Literature [14]	Cap et al.	Systematic review of TXA	2011	Favorable towards CRASH-2 findings and recommends use in combat situations and bleeding trauma patients

Tranexamic Acid and Trauma: Current Status and Knowledge Gaps with Recommended Research Priorities [15]	Pusateri et al., Department of Defense	Systematic review of TXA	2013	Identifies “knowledge gaps” in the CRASH-2 trial: (1) lack of clarity of reporting of monitoring, complications, (2) no transfusion benefit, and (3) data not robust

Tranexamic Acid in Trauma: How Should We Use It? [16]	Napolitano et al.	Systematic review of TXA	2013	Major knowledge gaps: (1) recruitment, (2) 100% follow-up, (3) 50% received transfusion, (4) survival benefit not a function of transfusion, (5) TBI greatest cause of death, (6) low rate of thrombosis, and (7) other shortcomings noted

**Table 3 tab3:** Nonrandomized controlled trials on TXA use in trauma.

Title	Author(s)	Description	Year	Outcomes	Recommendations
Military Application of Tranexamic Acid in Trauma Emergency Resuscitation (MATTERs) study [17]	Morrison et al.	Retrospective observational study; 896 pts with combat injury and at least one unit of PRBCs	2011	TXA significant survivor benefit with reduced blood utilization and increased rate of VTE	TXA should be incorporated into trauma resuscitation protocols for “severe wartime injury and hemorrhage”

Tranexamic Acid Use in Trauma: Effective but Not without Consequences [18]	Swendsen et al.	Retrospective multi cohort study; 126 trauma pts, 93 straight to OR/IR of whom 46 received TXA	2013	TXA mortality benefit, increased VTE, trend towards increased AKI, and no transfusions differences	“In civilian trauma, early TXA administration confers early survival advantage without affecting blood product usage but may increase the risk of DVT/PE and AKI”

Do All Trauma Patients Benefit from Tranexamic Acid? [19]	Valle et al.	Retrospective, observational single-center study; 1,217 trauma patients requiring OR/transfusions	2014	TXA group that had increased mortality, PRBCs, and crystalloid	“Prospective studies are needed to further identify conditions that may override the benefits from TXA”

Tranexamic Acid Use in Severely Injured Civilian Patients and the Effects on Outcomes [20]	Cole et al.	Prospective cohort study; 385 severely injured (ISS > 15), civilian pts. Focused on 131 shocked pts	2015	TXA mortality benefit for shocked patients not statistically significant; increased rate of VTE in TXA	TXA is recommended for “severely injured shocked patients”

**Table 4:** Editorials on TXA use.

Title	Author	Description	Year	Outcomes	Recommendations
CRASH-2 Goes Viral [21]	The Lancet	Editorial (Internet)	2011	Reviews CRASH-2 media coverage	Recommends TXA in trauma

Antifibrinolytics in trauma patients [24]	Kenji Inaba	Editorial (Internet/print)	2012	Reviews CRASH-2 MATTERs	Cites CRASH-2 “challenges” and notes MATTERs clarity

Trauma and Tranexamic Acid [22]	Gruen et al.	Editorial (Print)	2013	Reviews knowledge gaps noted by Napolitano et al. and Pusateri et al.	Recommends that TXA be confirmed in future RCTs

CRASH2, MATTERS…TXA in Trauma [23]	Mark Putland	Editorial (Internet/ blog)	2013	Reviews CRASH-2 MATTERs, MATTERs II	Data for seizures and thromboembolic complications remain incomplete. Needs more study.
